# Two New Reference Materials Based on Tobacco Leaves: Certification for over a Dozen of Toxic and Essential Elements

**DOI:** 10.1100/2012/216380

**Published:** 2012-03-12

**Authors:** Zbigniew Samczyński, Rajmund S. Dybczyński, Halina Polkowska-Motrenko, Ewelina Chajduk, Marta Pyszynska, Bożena Danko, Elżbieta Czerska, Krzysztof Kulisa, Katarzyna Doner, Paweł Kalbarczyk

**Affiliations:** Laboratory of Nuclear Analytical Methods, Institute of Nuclear Chemistry and Technology, Dorodna 16, 03-195 Warsaw, Poland

## Abstract

The preparation, certification, and characterization of two new biological certified reference materials for inorganic trace analysis have been presented. They are based on two different varieties of tobacco leaves, namely, Oriental Basma Tobacco Leaves (INCT-OBTL-5), grown in Greece, and Polish Virginia Tobacco Leaves (INCT-PVTL-6), grown in Poland. Certification of the materials was based on the statistical evaluation of results obtained in a worldwide interlaboratory comparison, in which 87 laboratories from 18 countries participated, providing 2568 laboratory averages on nearly 80 elements. It was possible to establish the certified values of concentration for many elements in the new materials, that is, 37 in INCT-OBTL-5 and 36 in INCT-PVTL-6, including several toxic ones like As, Cd, Hg, Pb, and so forth. The share and the role of instrumental analytical techniques used in the process of certification of the new CRMs are discussed.

## 1. Introduction

Health hazards associated with smoking of tobacco are known already for a long time and are well documented. According to World Health Organization, “Tobacco is the single most preventable cause of death in the world today. Unless urgent action is taken, tobacco could kill one billion people during this century. Tobacco is the only legal consumer product that can harm everyone exposed to it—and it kills up to half of those who use it as intended” [1]. As the recent study has shown, not only heavy smokers, but also persons smoking 1–4 cigarettes per day are exposed to a significantly higher risk of dying from ischemic heart disease and lung cancer than the nonsmokers [2]. Even nonsmokers may be affected; it is estimated that about 11% of all tobacco-related deaths are attributable to exposure to second-hand tobacco smoke [3].

Tobacco smoke is a toxic and carcinogenic mixture of more than 5000 chemicals [4]. From among 98 of most hazardous smoke components, 12% are metals and metalloids. As, Be, Cd, Cr (VI), Pb, and ^210^Po are carcinogens. Possible health hazards due to accumulation in the respiratory system of humans of alpha emitter ^210^Po (half-life 138.4 days) and its precursor ^210^Pb (half-life 22.2 years) were discussed by Skwarzec et al. [5]. Co and Se may cause problems with respiratory functions, Cu may adversely affect lung and immune system, Mn and Hg have an effect on nervous system, and Ni may cause chronic active inflammation and lung fibrosis [4].

It is perhaps worth mentioning that except of smoking tobacco health hazards to humans, the litter originating from smoking tobacco, that is, cigarette butts, if deposited into aquatic environment, may be acutely toxic both to marine and freshwater fish species [6]. The increasing pressure from international organizations as WHO is directed towards implementation of effective tobacco control measures in individual countries and promotion of smoke free environments [3]. The other way of diminishing health hazards might be the reduction of harmful components in tobacco. It is known that the content of metallic elements may vary from brand to brand [7–11]. Choosing a brand with a minimum content of harmful elements requires the use of reliable analytical techniques, and these in turn need a good system of analytical quality assurance. As is known, certified reference materials (CRMs) are the most widespread and relatively easy means of checking the accuracy of analytical methods [12, 13]. Best results can be expected when there is possibly close match of the CRM and test samples both in terms of general composition of the matrix and concentration levels of the analytes [14]. The only two CRMs with the tobacco leaf matrix that existed on the market, that is, Oriental Tobacco Leaves (CTA-OTL-1) [15] and Virginia Tobacco Leaves (CTA-VTL-2) [16], which gained great popularity among scientific community, were already exhausted. The present paper describes preparation and certification of two new CRMs with the tobacco leaf matrix, that is, Oriental Basma Tobacco Leaves (INCT-OBTL-5) and Polish Virginia Tobacco Leaves (INCT-PVTL-6). Each material was certified for more than 30 inorganic constituents, for several others information values were provided.

## 2. Preparation and Testing of the Materials

### 2.1. Origin, Preparation, and Homogenization of the Materials

**Figure 1:**
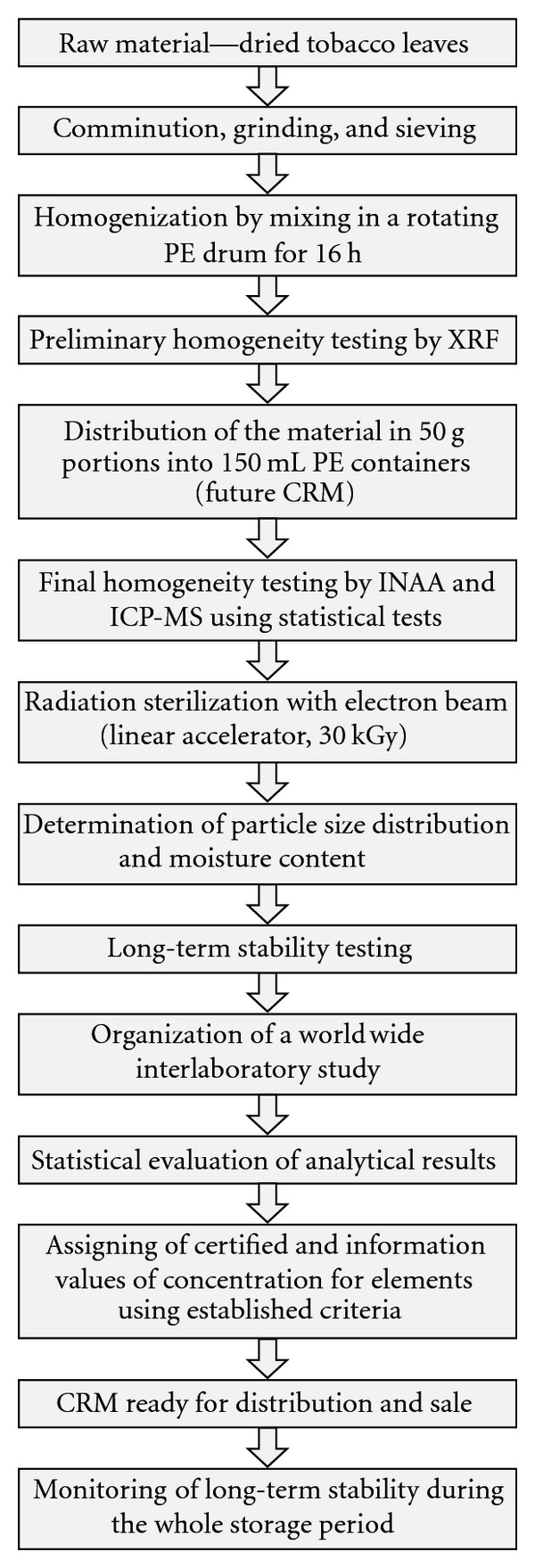
General strategy of production and certification of the new reference materials.

The candidate reference materials were prepared from dried tobacco leaves of two different varieties. Oriental Basma Tobacco Leaves (INCT-OBTL-5) was produced from tobacco grown in Greece, whereas Polish Virginia Tobacco Leaves (INCT-PVTL-6) from tobacco grown in Poland. An overview of the general strategy aimed at preparation and certification of new CRMs is presented in Figure 1. More details can be found in the published reports [17, 18]. Approximately, 48 kg of ground and sieved (through 100 *μ*m sieve) tobacco leaves powder of each variety was finally obtained. The whole lot of the given material was then homogenized by mixing for 16 hours. After this time, the preliminary homogeneity testing was carried out by determining content of selected elements using X-ray fluorescence (XRF) in several 5 g subsamples. The results confirmed that both Oriental Basma Tobacco Leaves and Polish Virginia Tobacco Leaves could be considered homogeneous. Consequently, the next step was their distribution in 50 g portions (future CRM) into polypropylene (PP) bottles. Furthermore, about 150 samples (c.a. 10 g) of each material were weighed (intercomparison samples). They were afterward sent to laboratories, which had declared their participation in this study. In order to ensure the long-term stability of the new CRMs, all containers with INCT-OBTL-5 and INCT-PVTL-6 were sterilized by electron beam radiation (energy 13 MeV, dose approx. 30 kGy) from a linear accelerator. The materials were next moved for storage in a special room air conditioned at 20°C.

### 2.2. Particle Size Determination

**Figure 2:**
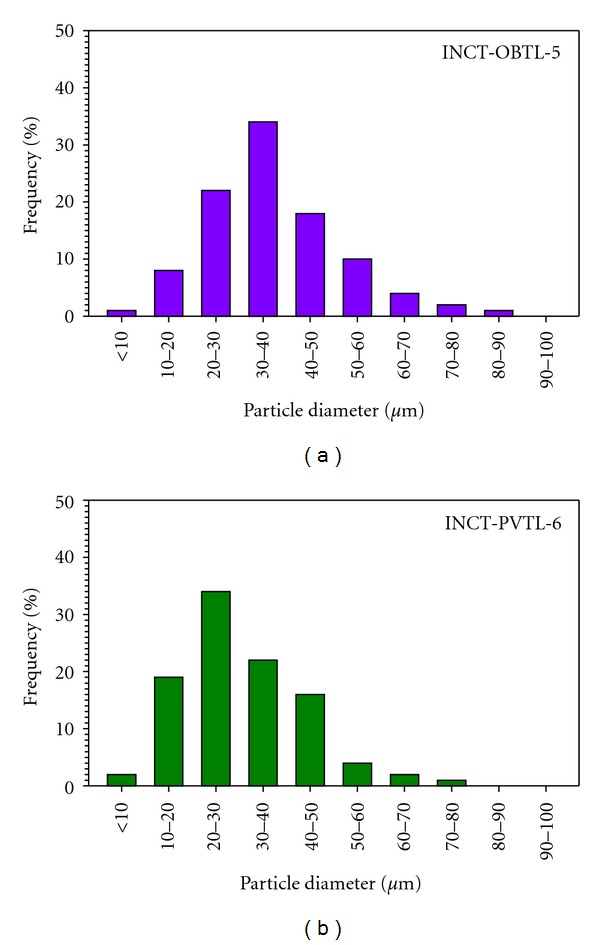
Particle size distribution of the candidate reference materials: Oriental Basma Tobacco Leaves (INCT-OBTL-5) and Polish Virginia Tobacco Leaves (INCT-PVTL-6).

Examination by optical microscopy was carried out in order to determine the particle size of the materials. Martin's diameter (arithmetic mean of the maximum distance between opposite sides of a particle and a distance in perpendicular direction) of 200 (randomly chosen) particles was determined. Results of the particle size analysis are presented in Figure 2. As can be seen, Martin's diameter below 60 *μ*m had over 90% particles. Distribution of the size was quite similar for INCT-OBTL-5 and INCT-PVTL-6 with a maximum in the range 30–40 and 20–30 *μ*m, respectively.

### 2.3. Moisture Content Determination

**Figure 3:**
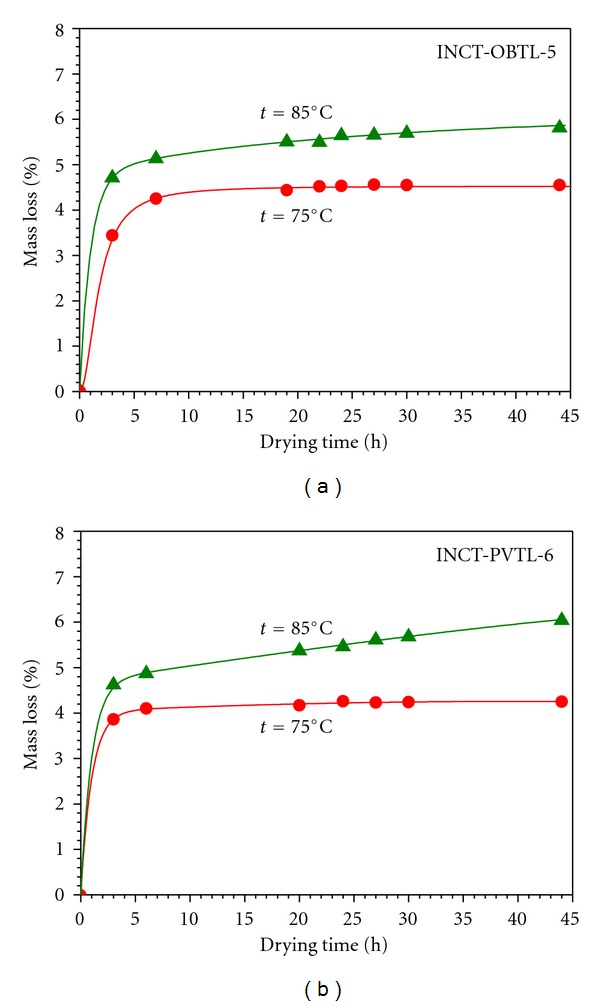
Water desorption curves for INCT-OBTL-5 and INCT-PVTL-6 at 75 and 85°C.

The moisture content may vary quite considerably with the changes in the ambient humidity and temperature. Therefore, concentration of elements in a given material must be always expressed on a dry-weight basis, irrespective of the actual water percentage in the sample. An obligation of great importance belonging to producers of reference materials is elaboration of reproducible procedure for the determination of dry mass. It was realized by establishing the water desorption curves for Oriental Basma Tobacco Leaves and Polish Virginia Tobacco Leaves separately at the temperature of 75 as well as 85°C. As follows from Figure 3, the water desorption curves for the temperature 75°C reach plateau after approx. 22 hours. The course of the dependence obtained for 85°C, however, might suggest that slight but discernible decomposition of the materials occurs. Losses of the mass were observed even at drying time exceeding 40 hours. Conclusions resulting from these studies made it possible to devise a recommended procedure for the determination of moisture content in INCT-OBTL-5 and INCT-PVTL-6. It consists in drying of the separate sample (not that taken for analysis) for 30 hours at 75°C.

### 2.4. Final Homogeneity Testing

Homogeneity is an absolutely obvious and indispensable feature of all reference materials. However, those of solid natural matrix are always heterogeneous on a microscopical scale, because they represent the population of particles varying in composition. In case of biological materials, nonuniform as a rule cell structure in various types of tissues and their subunits result in different elemental concentration [19]. Apparent homogeneity, that is, identical average composition of constituents for samples of a given mass is most often achieved by grinding and sieving followed by mixing of the material. Producers of CRMs are required to determine a minimum sample mass, for which a given material can be considered as homogeneous. Final homogeneity testing of INCT-OBTL-5 and INCT-PVTL-6 was performed for the nominal sample size of 100 mg concentration of Ce, Co, Cs, Eu, Fe, Hf, Rb, Tb in INCT-OBTL-5, and Ba, Co, Cs, Eu, Fe, Hf, Rb, Sc in INCT-PVTL-6 was determined applying Instrumental Neutron Activation Analysis (INAA). Six samples were taken from different six containers (chosen at random) from the whole population of containers, into which the given material was distributed. Analogously, six subsamples were taken from the seventh container randomly chosen as well. Homogeneity of the candidate reference materials was examined by statistical evaluation of the results obtained in the above two analytical series. The variances of determinations were compared by Fisher's test (*F*-test), whereas the means employing Student's *t*-test (*t*-test) [20]. As is evident from the data reported in Table 1, in case of all determined elements, the calculated parameters both *F* and *t* do not exceed the respective critical values *F*_0.95_ and *t*_0.05_. Hence, there are no significant differences in variances and means between the samples originating from different containers and those taken from a single container. A conclusion can be drawn that Oriental Basma Tobacco Leaves as well as Polish Virginia Tobacco Leaves can be considered as homogeneous for the sample masses greater than or equal to 100 mg. The other statistical approach employed for the final homogeneity checking was the analysis of variance (ANOVA) [21–24]. The content of Al, Ba, Cd, Co, Cr, Mg, Mn, Pb, Sb, Sr, V in INCT-OBTL-5, and Ba, Cd, Co, Eu, La, Li, Mg, Mn, Sr, V in INCT-PVTL-6 was determined using Inductively Coupled Plasma-Mass Spectrometry (ICP-MS). The obtained analytical results also fully confirmed good homogeneity of the both materials for the above sample size. According to the present recommendations to producers of CRMs, the standard uncertainty resulting from inhomogeneity of a given candidate reference material should be evaluated [21–24]. Its estimation is the so-called between-bottle variance, which can be calculated by means of the ANOVA method. The value of the uncertainty due to inhomogeneity amounted to 1.03% for INCT-OBTL-5 and 0.91% in case of INCT-PVTL-6.

### 2.5. Long-Term Stability Testing

Stability of future CRMs is also a parameter of essential importance. Statistical evaluation of data obtained from stability testing has two general purposes. The first is an assessment of the long-term stability of a given material and estimation of its shelf life. The second is an evaluation of the standard uncertainty connected to possible degradation during long-term storage, which should be included while calculating the combined standard uncertainties of certified values calculated later on [22, 24–30]. Long-term stability testing of Oriental Basma Tobacco Leaves and Polish Virginia Tobacco Leaves, stored under fixed and controlled conditions (20°C), covered a period of 22 months. After predetermined time intervals, samples of the given material were taken from one randomly chosen container. Concentration of six selected elements (Ce, Co, Fe, Rb, Sc, and Zn) was determined by INAA method. Assuming a linear regression model of degradation, stability testing data were fitted using the least square method [22, 26–28]:

(1) C=a+bx,

where *C* is the concentration, *x* is time, and *b* is the slope of the line (degradation rate). Of special importance in the stability studies is the standard deviation of the slope (*u*_*b*_) of the fitted regression line. The significance of the trend in the obtained results, which might hint at degradation of the material, can be assessed by comparing |*b* | /*u*_*b*_ to the value of a *t*-test (*α* = 0.05 and *n*–2 degrees of freedom) [27]. The course of the plots concentration versus time as well as statistical analysis of the calculated fitting parameters revealed no significant trends indicating instability of the candidate reference materials. The standard deviation of the slope of the obtained regression lines was used to estimate the standard uncertainty due to long-term stability [27]. Its value amounted to 0.61% for INCT-OBTL-5 and 0.67% for INCT-PVTL-6. The shelf life of the materials was established until the end of 2020. The stability of the new CRMs is planned to be monitored during the whole storage period.

## 3. Chemical Characterization of the Materials and Data Evaluation

### 3.1. Interlaboratory Comparison

A worldwide collaborative study on the determination of trace elements in Oriental Basma Tobacco Leaves (INCT-OBTL-5**) **and Polish Virginia Tobacco Leaves (INCT-PVTL-6) was organized. In this exercise, 87 participants from 18 countries took part contributing: 1318 laboratory averages (6092 individual determinations) on 78 elements for INCT-OBTL-5 and 1250 laboratory averages (5581 individual determinations) on 79 elements for INCT-PVTL-6 (see the appendix). The fundamental intention of the organizers was to certify the materials for possibly great number of elements, first of all those at trace level of concentration. The other important purpose of this study was also enabling the participants to compare their own results with those from other laboratories as well as with the finally established certified and/or information values.

The participants along with the candidate reference materials were requested to analyze also the provided reference material (RM), the identity of which was known to the organizers only. The results received for RM were employed in the further process of statistical evaluation of supplied data. Apart from purely analytical results, the participants were requested to report a short description about sample pretreatment, preconcentration and/or separation procedure (if any), and the technique of quantitative determination applied while analyzing the materials. On the basis of this information, the method symbol for each element was created in the special manner [17, 18]. To secure anonymity, the laboratories were coded, and exclusively the participant himself and the organizers have known the code number. All numbers reported in the report forms together with the respective laboratory codes and method symbols were entered into the specially constructed input file, separate for each of the material.

### 3.2. The Method of Data Evaluation

**Figure 4:**
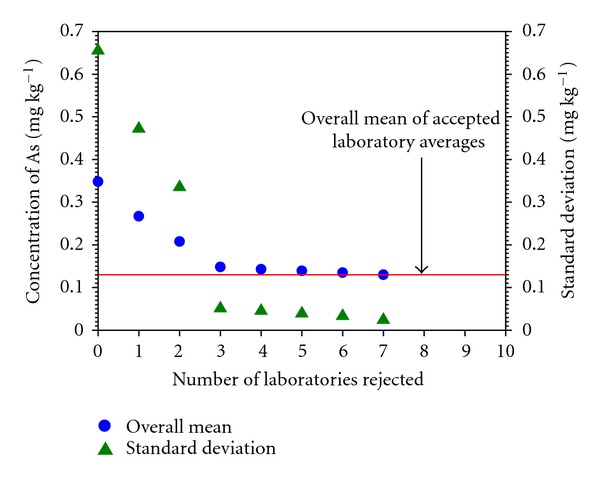
Changes of the overall mean and its standard deviation for As in INCT-PVTL-6 observed while executing the outlier's rejection procedure.

**Figure 5:**
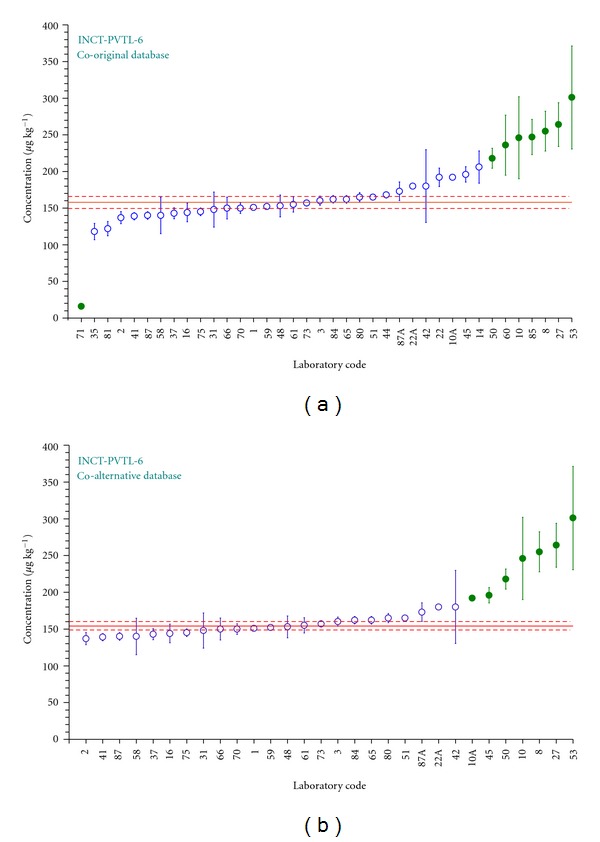
Z-shaped plots for Co in INCT-PVTL-6 while processing the original (a) and alternative databases (b). Laboratories qualified as outliers are marked with filled symbols; a solid horizontal line represents the overall mean and dashed lines confidence limits.

Evaluation of data supplied by laboratories participating in this exercise was performed employing the AQCS-1 software, dedicated to research groups dealing with certification of new CRMs [31]. The general idea of the program is the method of statistical data evaluation proposed by Dybczyński [32, 33], which is based on the outlier's rejection procedure using concurrently four statistical tests (Dixon, Grubbs, Skewness, and Kurtosis) at the significance level of 0.05. When a given laboratory average is classified as an outlier even by only one statistical test, it is removed from the population. The rejection process works until no further outlier is found, and then the final value of the overall mean for a given element is calculated together with the standard deviation, standard error, and confidence limits. A graphical illustration of how the process of outlier rejection affects the overall mean is demonstrated in Figure 4 on the example of results for As in INCT-PVTL-6. This method of data processing was applied in our laboratory during previous campaigns aimed at production of CRMs [34–37] and also by several other producers of CRMs [38–40]. For the purpose of this study, two input databases for each of the candidate reference materials were evaluated. The first called “original” contained all results for all elements supplied by participating laboratories. The second database called hereafter “alternative" was created from the original one, but only for those elements, however, for which the certified values in the reference material (sent to participants and analyzed by them along with INCT-OBTL-5 and INCT-PVTL-6) were available. The alternative database was formed as follows. Results delivered by every participant on a given certified element in the reference material (RM) were examined whether the confidence limits of the laboratory results at a significance level *α* = 0.05 overlapped with the confidence limit of the certified value. If not, the results of this laboratory for the considered element were removed from the original input file and the calculations were performed with the remaining data. The statistical evaluation of the both input files gave nearly in all cases very similar values of the overall mean as well as the confidence interval. As an illustration confirming this conclusion, the so-called Z-plots (a graphical representation of distribution of results) for Co in INCT-PVTL-6 are shown in Figure 5, representing the processing of the original (Figure 5(a)) and the alternative database (Figure 5(b)), respectively.

## 4. Certification of the Materials

### 4.1. Criteria for Assigning Certified and Information Values

The summary of the two intercomparisons organized in order to certify the candidate reference materials (in shortened version) is presented in Table 2. It gives a general overview on the content of determined elements in Oriental Basma Tobacco Leaves (INCT-OBTL-5**) **and Polish Virginia Tobacco Leaves (INCT-PVTL-6). The overall mean of concentration for any analyte, calculated as a result of a formal statistical evaluation of data supplied by participating laboratories, is still yet insufficient to be regarded as a certified value. This status can be granted only if definite criteria are fulfilled. Consideration whether or not a given “consensus” value can obtain the status of a certified value involves using certain, subjective, and thus necessarily arbitrary criteria. Such criteria established and thoroughly tested by us previously [33, 41] with some later modifications [34, 42, 43] are as follows.

1. The ratio of the one-sided confidence interval and the overall mean:
  (2) $$\frac{\text{SD}\;\cdot \;t_{0.05}}{\overset{®}{X}\;\cdot \;\sqrt{N}}\;\{\begin{array}{cc} \leq 20\text{\%} & \;\left(\text{trace}\;\text{elements}\right),\\ \leq 10\text{\%} & \;\left(\text{major}\;\text{elements}\right) \end{array}$$
  or relative standard deviation:
  (3) $$\frac{\text{SD}}{\overset{®}{X}}\{\begin{array}{cc} \leq 25\text{\%} & \;\left(\text{trace}\;\text{elements}\right),\\ \leq 15\text{\%} & \;\left(\text{major}\;\text{elements}\right), \end{array}$$
  where elements with concentration exceeding 5000 mg kg^−1^ (ppm) are considered to be the major elements.
2. The overall mean was calculated on the basis of at least four “accepted" laboratory means (*N* ≥ 4) obtained by more than one analytical technique. If results from only one analytical technique are available, the number of “accepted” laboratory averages used for the calculation of the overall mean cannot be smaller than five (*N* ≥ 5).
3. If the conditions (1) and (2) are fulfilled but the number of outliers exceeds 50%, the additional procedure is activated which repeats the process of outlier rejection from the beginning, checking simultaneously the changes of the mean and standard deviation accompanying successive rejections. The process of rejecting of outliers is then stopped when the successive change in both the mean and standard deviation becomes lower or equal to 15%. The condition (1) is then rechecked.
4. If the above criteria are met but there are indications that after outlier rejection performed on the whole population the remaining populations of results obtained by various analytical techniques differ significantly, the assignment of certified value is suspended.

The information values were assigned to those elements for which the results while not fulfilling simultaneously the conditions (1)–(4) still fulfilled the following condition:

(4) $$\frac{\text{SD}\;\cdot \;t_{0.05}}{\overset{®}{X}\;\cdot \;\sqrt{N}}\;\{\begin{array}{cc} \leq 50\text{\%} & \;\left(\text{trace}\;\text{elements}\right),\\ \leq 30\text{\%} & \;\left(\text{major}\;\text{elements}\right) \end{array}$$

calculated on the basis of at least three "accepted" laboratory averages and are quoted as numbers only, that is, without confidence intervals.

The elements, for which the obtained values did not fulfill the above criterion, were considered to be out of any classification.

### 4.2. Certified and Information Values

Applying the above-formulated criteria, the certified values of concentration were assigned to thirty-seven elements in Oriental Basma Tobacco Leaves (INCT-OBTL-5) *cf.* Table 3 and seventeen elements gained the status of information values (Table 4). In case of Polish Virginia Tobacco Leaves (INCT-PVTL-6), thirty-six elements could be certified (Table 5), and for thirteen analytes, it was possible to establish the information values (Table 6). The elements, for which the certified as well as information values were finally assigned as a result of statistical evaluation of the original or the alternative database, are accordingly marked in Table 2.

According to recent recommendations to CRM producers [22, 24, 25, 29], while calculating the combined standard uncertainty of the certified value *u*_c_, four contributions should be taken into account:

(5) $$u_{c}=\sqrt{u_{\text{interlab}}^{2}+u_{\text{lstab}}^{2}+u_{\text{inhom}}^{2}+u_{m}^{2}},$$

where *u*_interlab_ is estimated as standard deviation of the overall mean, *u*_lstab_ the standard uncertainty estimated from the long-term stability studies, *u*_inhom_ the standard uncertainty estimated from the homogeneity studies, and *u*_*m*_ the standard uncertainty due to moisture determination. The expanded uncertainty (*U*), corresponding to 95% confidence level, is obtained by multiplying *u*_c_ by a coverage factor *k* = *t*_0.05_ (Student's *t*-test parameter for *α* = 0.05 and *n*–1 degrees of freedom, where *n* is the number of laboratory averages). Certified (recommended) values are quoted together with their uncertainties ($\bar{X}\pm U$).

The metrological traceability is an important feature of CRMs [44]. In this study, the traceability of the new CRMs to the SI units was realized by the use of:

- RNAA ratio primary reference measurement procedures (RPRMP), (definitive methods),
- CRM sent by the interlaboratory comparison organizer and analyzed together with the candidate CRM,
- other CRMs chosen by participants,
- analytical methods calibrated against pure metals or oxides with full uncertainty budget.

**Figure 6:**
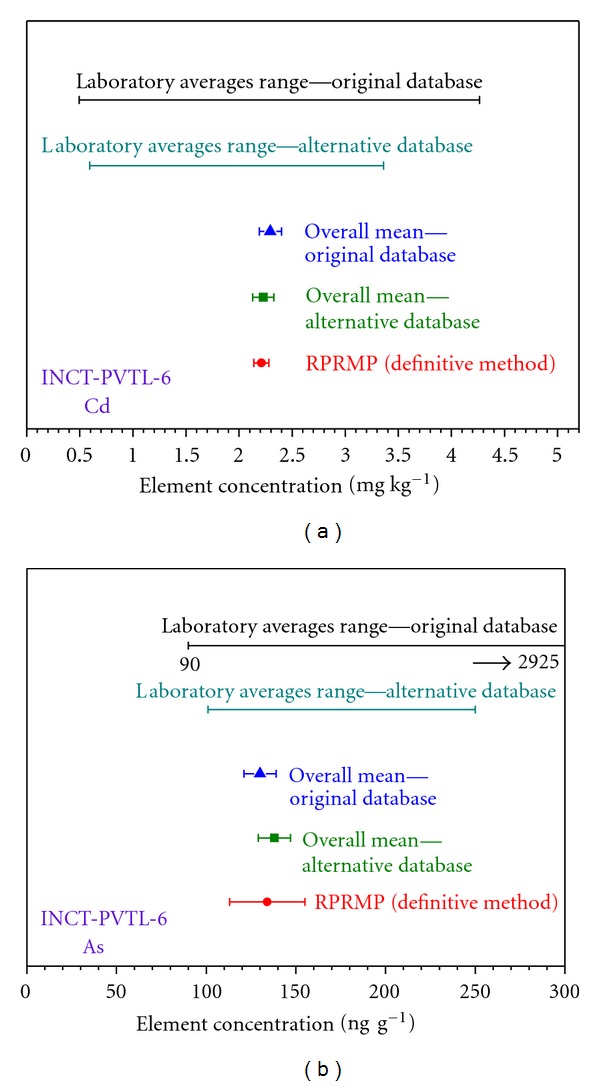
Comparison of the overall means and their confidence intervals for Cd and As in INCT-PVTL-6 obtained from the original and alterative databases with the result obtained by the definitive method and the ranges of laboratory averages.

The ratio primary reference measurement procedures (RPRMP) (definitive methods) with the highest metrological properties were developed in the Institute of Nuclear Chemistry and Technology [45–54]. They are based on quantitative and selective postirradiation radiochemical separation of the analyte of interest using ion exchange chromatography and/or extraction chromatography followed by gamma-ray spectrometric measurement [50–54]. The results of the determination of As and Cd by RPRMP (not included into the population of laboratory data) were applied for verification of the certified values *cf.* Figure 6. The values determined for Cd and As by the RPRMP are compared with the overall means calculated employing the original and alternative databases as well as with the corresponding ranges of laboratory averages sent by participants. One can note that the concentrations determined by means of RPRMP in INCT-PVTL-6 are in a very good agreement with the assigned certified values for As and Cd. Very similar picture was also observed in case of INCT-OBTL-5. Good agreement with the results obtained by the RPRMP methods prove additionally correctness of the certification procedure.

### 4.3. Some Remarks Concerning the Certification Process

**Figure 7:**
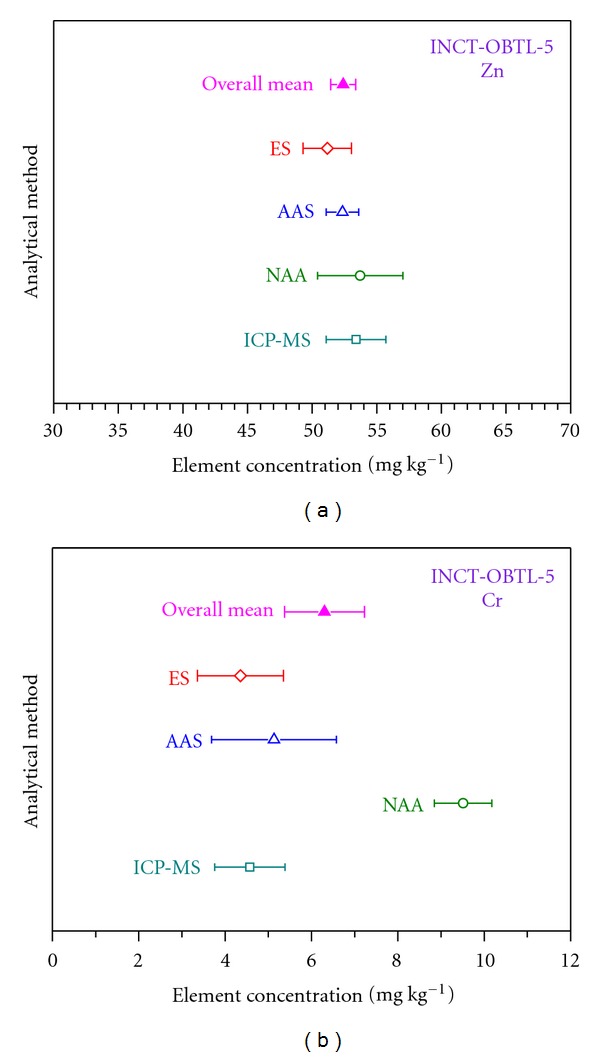
Comparison of the finally calculated overall mean (from the alternative database) for Zn and Cr in INCT-OBTL-5 and the means obtained for particular analytical techniques, together with the respective confidence intervals.

Observations and experiences gained during production and certification of our consecutive reference materials revealed a necessity to implement some improvements. Their general purpose was to prevent as much as possible establishing of certified values in doubtful situations. Of great importance is the fourth criterion concerning agreement of results on a given analyte produced by various analytical techniques. This problem is well illustrated in Figure 7, where the finally calculated overall means with their confidence intervals for zinc and chromium in INCT-OBTL-5 are confronted with the means and corresponding confidence limits obtained by NAA, ICP-MS, AAS (atomic absorption spectrometry), and ES (emission spectroscopy). The overall mean was calculated from the alternative input file. The individual mean values were computed employing appropriate input files, extracted from the initial dataset, which contained results determined using the given analytical method only. As is evident from Figure 7, in the case of zinc, there is a very good agreement among all individual techniques and the overall mean. Consequently, this element could be certified. Completely different conclusion can be drawn while considering the analogical graph for chromium. The mean established for NAA lies far from the values calculated for the rest of analytical methods as well as from the overall mean. Moreover, its confidence interval does not overlap with any other interval. Therefore, the status of the certified analyte could not be given in this case, despite the facts that the criteria 1–3 had formally been met and furthermore the number of laboratory averages was quite substantial (33) *cf.* Table 2. So, chromium has then been moved to the category of elements, for which only the information values are available. Analogous thorough examination underwent all other elements fully fulfilling the criteria 1–3 and thus potentially qualified as certified. Those, which finally gained this status, showed satisfactory agreement of analytical techniques.

Comparing the data included in Tables 3–6, one can easily note that the elements, which have gained the status of certified as well as information values, are very similar in both new CRMs. It should be born in mind, however, that the content of a given analyte is as a rule higher and in many cases even several times higher in Oriental Basma Tobacco Leaves compared to Polish Virginia Tobacco Leaves. The reason may most probably be, except of difference in variety, also significant differences in soil and climatic conditions, in which one and the other variety of tobacco leaves was grown (Greece and Poland). Such differences show the necessity of checking the elemental composition of tobacco in order to diminish the health hazards. On the other hand, distinctly different elemental composition in the new materials of similar biological matrix type is an interesting feature from the analytical point of view. It is worth emphasizing that the content of quite a number of elements regarded as rare and difficult in the analytical sense (e.g., Ag, Hf, Mo, Sc, Th, V, and most of rare earth elements (REE)) in both INCT-OBTL-5 and INCT-PVTL-6 could be certified. This remark concerns groups of elements classified as toxic (e.g., As, Cd, Hg, Pb) and essential (e.g., Co, Cu, and Zn) too. Some radioactive elements (^241^Am, ^137^Cs, ^40^K, ^210^Po, ^239^Pu, ^90^Sr, ^234^U, and ^238^U) were determined in the new CRMs as well. The population of results delivered for a given radionuclide was obviously too small (one or two laboratory averages) to establish any certified or information values. Nevertheless, the reported activities (Bq kg^−1^) of the mentioned isotopes in INCT-OBTL-5 and INCT-PVTL-6 may be useful for laboratories dealing with natural radioactivity measurements and for general assessment of health hazards due to smoking of tobacco.

### 4.4. Observations on Analytical Techniques

Apart from certification of new reference materials, the intercomparison provides valuable observations on analytical techniques as well. They concern individual role and position of particular techniques in the recent certification process, in comparison to earlier studies followed by drawing conclusions about outlined tendencies with the passage of time, deducing of future trends, and so forth.

**Figure 8:**
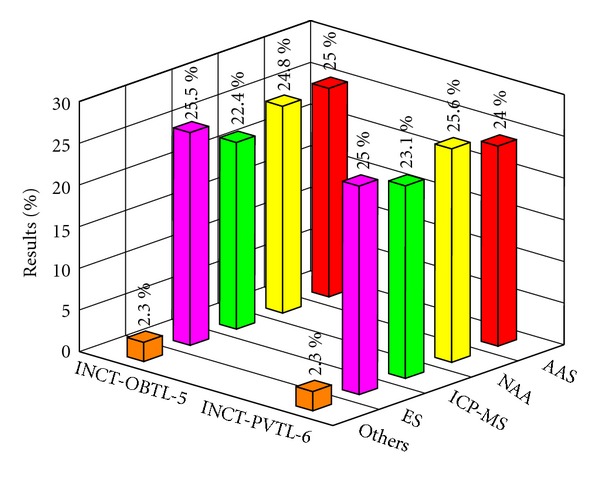
The share of analytical techniques in the certification process of INCT-OBTL-5 and INCT-PVTL-6.

Relative frequency of the use of particular analytical techniques in this study illustrates Figure 8. At the first glance, one can notice practically exclusive share of the four methods in the certification of Oriental Basma Tobacco Leaves, namely, NAA, AAS, ES, and ICP-MS. A novelty is their roughly balanced contribution to the population of results. This is a significant difference compared with our former exercises, when AAS and NAA distinctly prevailed [15, 37, 55]. Simultaneously, the frequency of the use of ES and especially ICP-MS has been increasing constantly and dynamically since the new millennium. As can be inferred, in the nearest future, they should attain a dominant position. The situation of still another technique, namely, XRF (X-ray Fluorescence), which supplied a moderate (a few percent) share of results in the past, is worth mentioning. Its contribution was diminishing over the past two decades. In this exercise, XRF has been completely absent.

The picture presented in Figure 8 reflects only the general share of analytical techniques in the whole population of the delivered data. The situation is somewhat different if particular analytes are considered individually. It is obvious that, due to limited sensitivity and/or susceptibility to various interferences, a given method is able to give reliable results only for the definite group of analytes depending on their level of concentration. Quite a number of elements were determined by at most two and even by a single analytical technique. Five elements, namely, Br (NAA), Er (ICP-MS), Hf (NAA), Sc (NAA), and Ta (NAA) were finally certified on the basis of only one method. A general conclusion can be drawn that NAA as well as ICP-MS are practically the only techniques employed for the determination of elements commonly regarded as rare and difficult from the analytical point of view, for example, REE, Ag, Hf, Mo, Ta, Tl, and so forth. It does not mean of course that share of these methods is less pronounced in other groups of analytes. An interesting phenomenon has been found in case of mercury, because its determination has been monopolized practically by one technique, that is, AAS in the cold vapor version. This observation is in agreement with conclusions resulting from the earlier studies, organized in the past decade. A certain measure of versatility of the given technique can also be the number of determined elements. The corresponding values are as follows: ICP-MS (68), NAA (44), ES (30), and AAS (22). One should bear in mind that the above observations do not necessarily reflect the real frequency of use of analytical techniques in every-day practice of laboratories as a whole. Nevertheless, they should be helpful in establishing of a general view on the analytical performance of individual methods.

## Figures and Tables

**Table:** (a)

Element (*γ* line, keV)	$\bar{x_{1}}\pm s_{1}$ mg kg^−1^	*n*	$\bar{x_{2}}\pm s_{2}$ mg kg^−1^	*m*	* F F*_0.05_	*t t*_0.05_
Ce (145.4)	3.09 ± 0.21	6	3.08 ± 0.36	6	2.098 < 5.050	2.068 < 2.228
Co (1332.5)	0.962 ± 0.028	6	0.991 ± 0.019	6	2.098 < 5.050	2.068 < 2.228
Cs (795.9)	0.239 ± 0.016	6	0.240 ± 0.013	6	1.626 < 5.050	0.186 < 2.228
Eu (344.3)	0.057 ± 0.004	6	0.058 ± 0.002	6	3.959 < 5.050	0.527 < 2.228
Fe (1291.6)	1534 ± 39	6	1572 ± 27	6	2.053 < 5.050	1.911 < 2.228
Hf (482.2)	0.258 ± 0.017	6	0.259 ± 0.011	6	2.393 < 5.050	0.182 < 2.228
Rb (1076.6)	17.2 ± 1.0	6	18.0 ± 0.5	6	4.853 < 5.050	1.728 < 2.228
Tb (879.4)	0.041 ± 0.011	6	0.032 ± 0.008	6	1.762 < 5.050	1.645 < 2.228

**Table:** (b)

Element (*γ* line, keV)	$\bar{x_{1}}\pm s_{1}$ mg kg^−1^	*n*	$\bar{x_{2}}\pm s_{2}$ mg kg^−1^	*m*	* F F*_0.05_	* t t*_0.05_
Ba (216.1)	37.1 ± 2.5	6	36.4 ± 2.9	6	1.321 < 5.050	0.408 < 2.228
Co (1332.5)	0.144 ± 0.005	6	0.145 ± 0.005	6	1.165 < 5.050	0.103 < 2.228
Cs (795.9)	0.023 ± 0.002	6	0.023 ± 0.003	6	1.472 < 5.050	0.186 < 2.228
Eu (344.3)	0.013 ± 0.002	6	0.013 ± 0.003	6	1.671 < 5.050	0.017 < 2.228
Fe (1291.6)	251 ± 8	6	257 ± 13	6	2.668 < 5.050	1.165 < 2.228
Hf (482.2)	0.139 ± 0.022	6	0.145 ± 0.021	6	1.129 < 5.050	0.480 < 2.228
Rb (1076.6)	5.78 ± 0.50	6	5.83 ± 0.32	6	2.509 < 5.050	0.221 < 2.228
Sc (889.3)	0.058 ± 0.004	6	0.059 ± 0.002	6	3.313 < 5.050	0.306 < 2.228

*F* = *s*_1(2)_^2^/*s*_2(1)_^2^, *F *_0.05_—critical value of the Fisher's test at significance level *α* = 0.05 and degrees of freedom *f*_1_ = *f*_2_ = 5.

$t=[\left|\bar{x_{1}}-\bar{x_{2}}\right|/\sqrt{\left(n-1\right)s_{1}^{2}\;+\;\left(m-1\right)s_{2}^{2}}\;]\cdot \sqrt{n\cdot m\;(n+m-2)/\left(n+m\right)}$, *t*_0.05_—critical value of the Student's *t*-test at significance level *α* = 0.05 and degrees of freedom *f* = *n* + *m*−2 = 10.

**Table 2:** Analytical results obtained for the candidate reference materials.

Element	Unit	Number of accepted laboratory averages		Overall mean of accepted laboratory averages	
		INCT-OBTL-5	INCT-PVTL-6	INCT-OBTL-5	INCT-PVTL-6
Ag	ng g^−1^	5	4	53.0^c1^	19.1^c1^
Al	mg kg^−1^	20	18	1981^c2^	252^c2^
^241^Am	Bq kg^−1^		1		0.022
As	ng g^−1^	19	19	668^c2^	138^c2^
Au	ng g^−1^	6	2	2.97^i1^	0.67
B	mg kg^−1^	13	13	33.6^c1^	33.4^c1^
Ba	mg kg^−1^	34	33	67.4^c2^	41.6^c2^
Be	ng g^−1^	8	7	81.2^i1^	31.0
Bi	ng g^−1^	2	3	96.0	140^i1^
Br	mg kg^−1^	11	10	87.4^c2^	19.5^c2^
C	wt%	1	1	44.0	42.9
Ca	wt%	42	42	3.996^c2^	2.297^c2^
Cd	mg kg^−1^	21	20	2.64^c2^	2.23^c2^
Ce	mg kg^−1^	15	16	2.99^c2^	0.743^c2^
Cl	wt%	4	4	0.772^i2^	0.457^i2^
Co	ng g^−1^	32	23	981^c2^	154^c2^
Cr	mg kg^−1^	33	30	6.30^i2^	0.911^i2^
Cs	ng g^−1^	19	12	288^c2^	26.6^i2^
^137^Cs	Bq kg^−1^	1	1	2.40	2.70
Cu	mg kg^−1^	50	47	10.1^c2^	5.12^c2^
Dy	ng g^−1^	5	2	184^i1^	38.0
Er	ng g^−1^	5	5	101^c1^	18.5^c1^
Eu	ng g^−1^	17	14	60.2^c2^	14.0^c2^
F	mg kg^−1^	1	1	26.4	19.6
Fe	mg kg^−1^	60	61	1491^i1^	258^i1^
Ga	ng g^−1^	2	2	518	78
Gd	ng g^−1^	3	2	243^i1^	52
Ge	ng g^−1^	2	1	34	10
Hf	ng g^−1^	10	9	291^c1^	161^c1^
Hg	ng g^−1^	20	23	20.9^c2^	23.2^c2^
Ho	ng g^−1^	3	1	34.5^i1^	6.0
I	ng g^−1^	3	2	843	337
In	ng g^−1^	1	1	2.0	0.5
K	wt%	41	40	2.271^c2^	2.640^c2^
^40^K	Bq kg^−1^	1	1	633	722
La	mg kg^−1^	16	16	1.69^c2^	0.540^c2^
Li	mg kg^−1^	11	10	19.3^i1^	3.35^c1^
Lu	ng g^−1^	6	4	16.7^i2^	5.7
Mg	wt%	37	37	0.853^c2^	0.241^c2^
Mn	mg kg^−1^	43	43	180^c2^	136^c2^
Mo	ng g^−1^	8	7	414^c1^	396^c1^
N	wt%	2	2	2.7	2.8
Na	mg kg^−1^	21	21	435^i2^	62.4^i2^
Nb	ng g^−1^	2	2	127	33
Nd	mg kg^−1^	6	5	1.33^c1^	0.322^c1^
Ni	mg kg^−1^	24	21	8.50^c2^	1.49^c2^
P	wt%	16	17	0.170^c1^	0.242^c1^
Pb	mg kg^−1^	20	19	2.01^c2^	0.972^c2^
Pd	ng g^−1^		1		45
^210^Po	Bq kg^−1^	2	2	19.5	22.2
Pr	ng g^−1^	4	4	321^i1^	82.9^i1^
Pt	ng g^−1^	1	1	0.1	0.1
^239^Pu	Bq kg^−1^	1		0.007	
Rb	mg kg^−1^	18	17	19.1^c^	5.97^c^
Re	ng g^−1^	1	1	7.0	1.4
Ru	ng g^−1^	1	1	0.8	1.0
S	wt%	6	6	0.455^c2^	0.378^c2^
Sb	ng g^−1^	12	10	75.5^c1^	37.2^c1^
Sc	ng g^−1^	10	11	640^c2^	59.5^c2^
Se	ng g^−1^	8	11	172	153
Si	wt%	5	5	0.527	0.198
Sm	ng g^−1^	16	14	264^c2^	58.0^c2^
Sn	ng g^−1^	5	3	260	31.1^i1^
Sr	mg kg^−1^	24	24	105^c2^	133^c2^
^90^Sr	Bq kg^−1^	1	1	21	13
Ta	ng g^−1^	5	5	41.7^c1^	10.9^c1^
Tb	ng g^−1^	8	7	34.7^c2^	8.1^c2^
Th	ng g^−1^	11	11	503^c2^	88.8^c2^
Ti	mg kg^−1^	11	11	80.7^i1^	12.3^i1^
Tl	ng g^−1^	4	3	51.3^i2^	22.8^i2^
Tm	ng g^−1^	3	2	13.6^i1^	2.4
U	ng g^−1^	13	7	113^i1^	22.0^i1^
^234^U	Bq kg^−1^	1	1	0.51	0.34
^238^U	Bq kg^−1^	2	2	1.24	0.92
V	mg kg^−1^	10	10	4.12^c2^	0.405^c2^
W	ng g^−1^	1	2	33	32
Y	ng g^−1^	4	4	963^i1^	218^i1^
Yb	ng g^−1^	10	7	115^c2^	28.3^i2^
Zn	mg kg^−1^	64	64	52.4^c2^	43.6^c2^
Zr	mg kg^−1^	7	7	6.35	3.50

^c1^Certified value assigned employing the original database, ^c2^employing the alternative database. ^i1^Information value assigned employing the original database, ^i2^employing the alternative database.

**Table:** (a) Major and minor elements

Element	Concentration (wt%)
Al	0.198 ± 0.028
Ca	3.996 ± 0.142
K	2.271 ± 0.076
Mg	0.853 ± 0.034
P	0.170 ± 0.012
S	0.455 ± 0.091

**Table:** (b) Trace elements (1–1000 mg kg^−1^)

Element	Concentration (mg kg^−1^)
B	33.6 ± 2.2
Ba	67.4 ± 3.8
Br^a^	87.4 ± 5.4
Cd	2.64 ± 0.14
Ce	2.99 ± 0.18
Cu	10.1 ± 0.4
La	1.69 ± 0.09
Mn	180 ± 6
Nd	1.33 ± 0.11
Ni	8.50 ± 0.49
Pb	2.01 ± 0.31
Rb	19.1 ± 1.0
Sr	105 ± 5
V	4.12 ± 0.55
Zn	52.4 ± 1.8

**Table:** (c) Trace elements (below 1 mg kg^−1^)

Element	Concentration (ng g^−1^)
Ag	53.0 ± 10.5
As	668 ± 86
Co	981 ± 67
Cs	288 ± 20
Er^a^	101 ± 6
Eu	60.2 ± 4.1
Hf^a^	291 ± 24
Hg	20.9 ± 1.3
Mo	414 ± 62
Sb	75.5 ± 12.5
Sc^a^	640 ± 27
Sm	264 ± 13
Ta^a^	41.7 ± 3.8
Tb	34.7 ± 2.3
Th	503 ± 43
Yb	115 ± 23

^a^Certified on the basis of results by single analytical method.

**Table 4:** Information values for INCT-OBTL-5.

Element	Concentration	Unit
Au	3.0	ng g^−1^
Be	81.2	ng g^−1^
Cl	0.772	wt%
Cr	6.30	mg kg^−1^
Dy	184	ng g^−1^
Fe	0.149	wt%
Gd	243	ng g^−1^
Ho	34.5	ng g^−1^
Li	19.3	mg kg^−1^
Lu	16.7	ng g^−1^
Na	435	mg kg^−1^
Pr	321	ng g^−1^
Ti	80.7	mg kg^−1^
Tl	51.3	ng g^−1^
Tm	13.6	ng g^−1^
U	113	ng g^−1^
Y	963	ng g^−1^

**Table:** (a) Major and minor elements

Element	Concentration (wt%)
Ca	2.297 ± 0.078
K	2.640 ± 0.090
Mg	0.241 ± 0.009
P	0.242 ± 0.015
S	0.378 ± 0.059

**Table:** (b) Trace elements (1–1000 mg kg^−1^)

Element	Concentration (mg kg^−1^)
Al	252 ± 49
B	33.4 ± 1.9
Ba	41.6 ± 1.9
Br^a^	19.5 ± 1.0
Cd	2.23 ± 0.12
Cu	5.12 ± 0.20
Li	3.35 ± 0.67
Mn	136 ± 5
Ni	1.49 ± 0.14
Rb	5.97 ± 0.28
Sr	133 ± 6
Zn	43.6 ± 1.4

**Table:** (c) Trace elements (below 1 mg kg^−1^)

Element	Concentration (ng g^−1^)
Ag	19.1 ± 3.8
As	138 ± 10
Ce	743 ± 51
Co	154 ± 7
Er^a^	18.5 ± 3.2
Eu	14.0 ± 2.6
Hg	23.2 ± 1.6
Hf^a^	161 ± 8
La	540 ± 27
Mo	396 ± 29
Nd	322 ± 24
Pb	972 ± 147
Sb	37.2 ± 3.9
Sc^a^	59.5 ± 3.4
Sm	58.0 ± 4.3
Ta^a^	10.9 ± 1.2
Tb	8.1 ± 1.0
Th	88.8 ± 6.8
V	405 ± 56

^a^Certified on the basis of results by single analytical method.

**Table 6:** Information values for INCT-PVTL-6.

Element	Concentration	Unit
Bi	140	ng g^−1^
Cl	0.457	wt%
Cr	911	ng g^−1^
Cs	26.6	ng g^−1^
Fe	258	mg kg^−1^
Na	62.4	mg kg^−1^
Pr	82.9	ng g^−1^
Sn	31.1	ng g^−1^
Ti	12.3	mg kg^−1^
Tl	22.8	ng g^−1^
U	22.0	ng g^−1^
Y	218	ng g^−1^
Yb	28.3	ng g^−1^

## Appendix

### List of Participants of the Intercomparison INCT-OBTL-5 and INCT-PVTL-6 (in Alphabetical Order)

Mr. A. Barzev, CSIR-National Metrology Laboratory, Pretoria, South Africa; Mr. L. Bednarzak M.Sc., Powiatowa Stacja Sanitarno-Epidemiologiczna, Siedlce, Poland; Mr. L. Bielawski M.Sc., Uniwersytet Gdański, Gdańsk, Poland; Mrs. J. Biel-Ćwikowska M.Sc., Wojewódzki Inspektorat Ochrony Środowiska we Wrocławiu, Delegatura w Jeleniej Górze, Poland; Dr. A. Bielicka, Uniwersytet Gdański, Gdańsk, Poland; Mr. B. Blazhev, Ministry of Agriculture and Food Supply, Central Laboratory for Chemical Testing and Control, Sofia, Bulgaria; Mrs. G. Boguszewicz M.Sc., Mrs. E. Wiśniewska, Wojewódzka Stacja Sanitarno-Epidemiologiczna, Olsztyn, Poland; Prof. Dr. M.H. Borawska, Dr. K. Socha, Mrs. J. Soroczyńska M.Sc., Akademia Medyczna, Białystok, Poland; Mr. O. Boyum, Norwegian Medicines Agency, Oslo, Norway; Mrs. J. Charasińska M.Sc., Polskie Odczynniki Chemiczne S.A., Gliwice, Poland; Dr. E. Cortes Toro, Mrs. X. Rojas, Mr. M. Sepulveda, Comision Chilena De Energia Nuclear, Santiago de Chile, CHILE; Mrs. M. Czapczyk M.Sc., Wojewódzka Stacja Sanitarno-Epidemiologiczna, Warszawa, Poland; Dr. D. Danisiewicz-Czupryńska, Uniwersytet Gdański, Gdańsk, Poland; Dr. B. Danko, Mr. K. Kulisa, Dr. Z. Samczyński, Instytut Chemii i Techniki Jądrowej, Warszawa, Poland; Mrs. G. Dembska, Ł. Zegarowski, Instytut Morski, Gdańsk, Poland; Dr. A. Drzewińska, Wojskowa Akademia Techniczna, Warszawa, Poland; Prof. Dr. J. Falandysz, Uniwersytet Gdański, Gdańsk, Poland; Prof. Dr. Z. Fijałek, Mrs. M. Kiljan, Narodowy Instytut Leków, Warszawa, Poland; Dr. M. C. Freitas, Dr. H. M. Dung, Mrs. I. Dionisio, Institute for Technology and Nuclear, Sacavem, Portugal; Mrs. R. Georgiewa, Mrs. L. Metchkueva, Mrs. M. Bakardjieva, Mrs. D. Stankova, Ministry of Heath, Element Composition Laboratory, Sofia, Bulgaria; Prof. Dr. J. Golimowski, Uniwersytet Warszawski, Warszawa, Poland; Mrs. W. Gołębiewska M.Sc., Mennica Metale Szlachetne S.A., Warszawa, Poland; Dr. H. Górecka, Mrs. S. Biegańska M.Sc., Mrs. M. Barańska M.Sc., Politechnika Wrocławska, Wrocław, Poland; Mr. A. Hryniszyn M.Sc., Instytut Metali Nieżelaznych, Gliwice, Poland; Dr. R. Jacimovic, Josef Stefan Insitute, Ljubljana, Slovenia; Mr. A. Jaklewicz M.Sc., Akademia Medyczna, Warszawa, Poland; Mrs. A. Jelińska M.Sc., Mrs. M. Miśkiewicz, Mrs. J. Majchrowicz, Wojewódzki Inspektorat Weterynarii, Gdańsk, Poland; Mr. Z. Kaliszewski M.Sc., Wojewódzki Inspektorat Ochrony Środowiska w Białymstoku, Poland; Dr. V. K. Karandashev, Institute of Microelectronics Technology and High Purity Materials, Russian Academy of Sciences, Moscow, Russia; Mrs. D. Karmasz M.Sc., Mrs. E. Górecka M.Sc., Państwowy Instytut Geologiczny, Warszawa, Poland; Mr. P. Knapik, Instytut Metalurgii Żelaza, Gliwice, Poland; Mrs. D. Kolasa M.Sc., Mrs. B. Arndt M.Sc., Instytut Chemii Przemysłowej, Warszawa, Poland; Dr. P. Konieczka, Mrs. A. Beyer M.Sc., Prof. Dr. J. Namieśnik, Politechnika Gdańska, Gdańsk, Poland; Dr. W. Korol, Mrs. H. Nieściór M.Sc., Mrs. D. Kuśnierz M.Sc., Mrs. A. Zniszczyńska M.Sc., Instytut Zootechniki – PIB, Krajowe Laboratorium Pasz, Lublin, Poland; Mrs. A. Korona M.Sc., Powiatowa Stacja Sanitarno-Epidemiologiczna, Wałbrzych, Poland; Dr. R. Kowalski, Akademia Rolnicza, Lublin, Poland; Mrs. R. Kozioł M.Sc., Zakłady Azotowe w Tarnowie-Mościcach S.A., Poland; Mrs. A. Kwiatkowska M.Sc., Mrs. A. Staniszewska M.Sc., Mr. J. Sikora M.Sc., Wojewódzki Inspektorat Weterynarii, Szczecin, Poland; Mr. C. Legutko M.Sc., Wojewódzki Inspektorat Ochrony Środowiska we Wrocławiu, Delegatura w Wałbrzychu, Poland; Mrs. A. Lerska M.Sc., J. S. Hamilton Poland Sp. z. o.o., Gdynia, Poland; Dr. K. Loska, Dr. D. Wiechuła, Dr. I. Korus, Politechnika Śląska, Gliwice, Poland; Dr. S. M. Lyapunov, Mrs. O. Okina, Mr. A. Gorbunov, Geological Institute, Moscow, Russia; Mrs. B. Łysiak-Konarzewska M.Sc., Dr. M. Karger, Mrs. S. Lamarche, Mrs. A. Dobrowolska, Uniwersytet Warszawski, Warszawa, Poland; Mr. K. Maciołek M.Sc., Aquanet S. A., Poznań, Poland; Mrs. M. Majcherek M.Sc., Mrs. J. Olesik M.Sc., Okręgowa Stacja Chemiczno-Rolnicza, Łódź, Poland; Prof. Dr. H. Matusiewicz, Politechnika Poznańska, Poznań, Poland; Mrs. Z. Mazurek M.Sc. Okręgowa Stacja Chemiczno-Rolnicza, Warszawa, Poland; Dr. V. G. Merkulov, Mr. D.V. Kabanov, Tomsk Polytechnic University, Tomsk, Russia; Dr. T. van Merteen, Delft University of Technology, Delft, The Netherlands; Mr. P. Mierzwiński M.Sc., Główny Instytut Górnictwa, Katowice, Poland; Dr. J. Mietelski, Instytut Fizyki Jądrowej, Kraków, Poland; Dr. B. Mikuła, Dr. A. Kita, Prof. Dr. F. Buhl, Uniwersytet Śląski, Katowice, Poland; Prof. Dr. E. A. De Nadai Fernandes, Dr. M. A. Bacchi, Mr. C. L. Gonzaga, Centro de Energia Nuclear na Agricultura, Universidade de Sao Paulo, Sao Paulo, Brazil; Dr. K. Oprządek, Mrs. B. Łopuszyńska M.Sc., Akademia Podlaska, Siedlce, Poland; Dr. A. Pantelica, National Institute of Phisics and Nuclear Engineering “Horia Hulubei”, Bucharest, Romania; Mrs. M. Pietrasik M.Sc., Wojewódzka Stacja Sanitarno-Epidemiologiczna w Rzeszowie, Oddział Laboratoryjny w Tarnobrzegu, Poland; Dr. Shri V. D. Puranik, Dr. G. G. Pandit, Bhabha Atomic Research Centre, Mumbai (Bombay), India; Dr. L. Quanwei, China Institute of Atomic Energy, Beijing China; Dr. M. Rajkowska, Akademia Rolnicza, Szczecin, Poland; Dr. K. Ratajczak, Wojewódzki Inspektorat Ochrony Środowiska, Poznań, Poland; Dr. W. Reczyński, Akademia Górniczo-Hutnicza, Kraków, Poland; Dr. S. Resnizky, Mr. R. Invernizzi, Comision Nacional de Energia Atomica, Buenos Aires, Argentina; Mrs. M. Rybołowicz M.Sc., Mrs. R. Borkowska M.Sc., “Herbapol-Lublin” S. A., Oddział w Białymstoku, Poland; Dr. M. Saiki, Mr. E. R. Alves, Instituto de Pesquisas Energéticas e Nucleares, Sao Paulo, Brazil; Prof. Dr. B. Skwarzec, Uniwersytet Gdański, Gdańsk, Poland; Dr. M. Smolik, Zakład Higieny Weterynaryjnej, Gorzów Wielkopolski, Poland; Mrs. K. Starska M.Sc., Państwowy Zakład Higieny, Warszawa, Poland; Prof. E. Steinnes, Mr. S. Lierhagen, Norwegian University of Science and Technology, Trondheim, Norway; Dr. J. H. Sterba, Mr. M. Bichler, Vienna University of Technology, Austria; Prof. Dr. P. Szefer, Mrs. M. Misztal-Szkudlińska M.Sc., Dr. M. Grembecka, Akademia Medyczna, Gdańsk, Poland; Dr. J. Szkoda, Państwowy Instytut Weterynaryjny, Puławy, Poland; Mrs. U. Szuberla M.Sc., Mrs. T. Kucharska M.Sc., Akademia Rolnicza, Szczecin, Poland; Dr. I. Szynkowska, Dr. E. Leśniewska, Mrs. J. Albińska M.Sc., Dr. A. Pawlaczyk, Politechnika Łódzka, Łódź, Poland; Mr. M. Szypuła M.Sc., “ENERSYS” Sp. z o.o., Bielsko-Biała, Poland; Mr. Z. Trela, Mr. Ł. Cnota M.Sc., Mr. A. Grabowski, Ośrodek Badań i Kontroli Środowiska, Katowice, Poland; Mrs. B. Tryba M.Sc., Wojewódzki Inspektorat Ochrony Środowiska w Krakowie, Delegatura w Tarnowie, Poland; Mrs. Maria Turkowska, Mrs. E. Szymańska, Mrs. M. Matuszczyk, Mrs. M. Kaczmarzyk, Wojewódzka Stacja Sanitarno-Epidemiologiczna, Kielce, Poland; Dr. Z. Varga, Hungarian Academy of Sciences, Budapest, Hungary; Ing. P. Vermaercke, Belgian Nuclear Research Centre, Mol, Belgium; Dr. S. Walas, Mrs. H. Mrowiec M.Sc., Uniwersytet Jagielloński, Kraków, Poland; Dr. Wei-Yue Feng, Institute of High Energy Physics, Chinese Academy of Sciences, Beijing China; Dr. M. Wełna, Dr. J. Borkowska-Burnecka, Prof. Dr. W. Żyrnicki, Politechnika Wrocławska, Wrocław, Poland; Mrs. E. Węgrzyn M.Sc., Mrs. E. Jarocka M.Sc., Szkoła Główna Gospodarstwa Wiejskiego, Warszawa, Poland; Dr. M. Wojtczak, Mr. Z. Tamborski M.Sc., Politechnika Łódzka, Łódź, Poland; Prof. Dr. A. M. Yusof, Universiti Teknologi Malaysia, Skudai, Malaysia; Dr. P. Zagrodzki, Mr. M. Szałkowski M.Sc., Instytut Fizyki Jądrowej PAN, Kraków, Poland; Mrs. D. Żurawska M.Sc., Mrs. W. Olech, Graniczna Stacja Sanitarno-Epidemiologiczna, Elbląg, Poland.
